# Empirical and Theoretical Bases of Good Steamed Bread Production

**DOI:** 10.3390/foods12030433

**Published:** 2023-01-17

**Authors:** Yanchun Peng, Yun Zhao, Xiaojie Jin, Yin Xiong, Jing Dong, Wujun Ma

**Affiliations:** 1 College of Agronomy, Qingdao Agricultural University, Qingdao 266109, China; 2 Hubei Key Laboratory of Food Crop Germplasm and Genetic Improvement, Institute of Food Crops, Hubei Academy of Agricultural Sciences, Wuhan 430064, China; 3 Institute of Cereal and Oil Crops, Hebei Academy of Agriculture and Forestry Sciences, Hebei Provincial Laboratory of Crop Genetics and Breeding, Shijiazhuang 050035, China; 4 National R&D Center for Se-rich Agricultural Products Processing, School of Modern Industry for Selenium Science and Engineering, Wuhan Polytechnic University, Wuhan 430023, China; 5 Food Futures Institute, College of Science, Health, Engineering and Education, Murdoch University, Perth 6150, Australia

**Keywords:** Chinese steamed bread, wheat gluten proteins, glutenins, gliadins, rheological properties, frozen storage

## Abstract

Chinese steamed bread (CSB) is a main staple food in China, accounting for 40% of wheat flour usage in China. Due to its health benefits, CSB is gaining popularity across the world. In this review, the effects of gluten proteins (particularly glutenins and gliadins) on the quality of CSB are summarized from the literature. Requirements of appropriate rheological parameters in different studies are compared and discussed. Along with the increasing demand for frozen storage food, there are obvious increases in the research on the dynamics of gluten proteins in frozen dough. This review also summarizes the factors influencing the deterioration of CSB dough quality during frozen storage as well as effective measures to mitigate the negative effects.

## 1. History and Categorizations of Chinese Steamed Bread

China is the largest wheat (*Triticum aestivum* L.) producer in the world, with a cultivation area of over 20 million ha and a production of over 110 million tons (23.38 million ha and 134.25 million tons in 2020, data from national bureau of statistics, http://www.stats.gov.cn/, accessed on 5 March 2020). Chinese steamed bread (CSB) is a traditional and main staple food in China. In northern China, around 70% of wheat flour is used for CSB production; on average, CSB accounts for approximately 40% of the total wheat consumption in China [1,2]. CSB originated in the Three Kingdoms period, and can be dated back to 1700 years ago, with the peak development in the Song Dynasty and Yuan Dynasty [3]. However, despite such a long history, the production of CSB remains the same. The name ‘steamed bread’ is derived from the method of cooking fermented dough by steaming. The traditional method of making CSB contributes to the unique flavor, pleasant aroma and smooth white skin of the product, making it distinct from Western bread [3]. In China, CSB is more popular in the northern region than in the south. It is also called ‘Oriental bread’. The production of CSB only requires the ingredients of wheat flour, water and yeast or sourdough, except for the Guangdong-style CSB, in which some additional ingredients such as fat and sugar are usually incorporated [4,5,6]. Because the production process of CSB involves steaming instead of baking, CSB generally has a significantly lower level of acrylamide, which is a detrimental and carcinogenic substance for humans [7]. Additionally, because of the preservation of more nutrients under the relatively low temperature of steaming and the low incorporation of oil and salt, CSB is considered healthier than Western bread [8].

Generally, CSB is categorized into three types: northern style, southern style and Guangdong style. Northern-style CSB is generally characterized by high cohesiveness and elasticity with a white smooth skin, dense texture and bland taste, while southern-style CSB is of low cohesiveness and a soft and open texture with white skin and a sweet taste [4,9]. Guangdong-style CSB is generally consumed as a type of dessert in various forms [5]. Northern-style CSB is mainly consumed in northern China, while southern-style and Guangdong-style CSB are consumed not only in southern China, but also in Japan, Korea and some Southeast Asian countries [1,10]. Due to its simpler formula, the quality and appearance of northern-style CSB are more easily affected by the different classifications of wheat varieties [11].

## 2. Wheat Gluten Proteins and Their Effects on CSB Production

### 2.1. Characteristics of Gluten Proteins in Wheat Dough

Wheat gluten proteins account for more than 85% of the total wheat grain proteins and are responsible for the viscoelastic properties of wheat dough [12,13]. During the formation process of dough, the two major kinds of gluten proteins, glutenins and gliadins, interact with each other to form a viscoelastic three-dimensional network, which is stabilized mainly by covalent disulfide (SS) bonds and known as gluten [14]. Dough properties such as strength, extensibility and stability are critical for the quality of dough. These properties are dependent on the structure and composition of gluten proteins, which in turn determine the end use of wheat flour for flour-based foods and their quality [9,15,16].

Several different chemical bonds are involved in the formation of the gluten network and dough structure. Among them, SS bonds can form strong cross-links within and between polypeptide chains to stabilize hydrogen bonds and hydrophobic interactions, and thereby play a key role in dough formation and development [17]. During this process, free sulfhydryl (SH) bonds participate in the interaction with SS bonds, leading to the formation and rearrangement of large protein aggregates known as glutenin macropolymer (GMP) and also referred to as SDS-unextractable polymeric protein (UPP) [18,19]. Cysteine residues distributed in glutenins and gliadins are the bases for the formation of these SS bonds, and therefore more free cysteine residues may result in stronger wheat gluten [20]. The proportion of UPP is recognized as a determinant of dough strength [12,21], and is correlated with the total score of Guangdong- and northern-style CSB [22]. A study on effects of adding selectively hydrolyzed soy protein (SHSP) to CSB quality suggested that 1% SHSP addition could enhance the GMP by 7.90% and decrease the contents of β-turns, which resulted in higher loaf volume, lower hardness and higher viscoelasticity [23]. Thus, SHSP is popularly used for CSB production, especially for wheats with relatively lower protein contents [24]. The amount of UPP is more strongly influenced by the genotype, whereas that of extractable polymeric proteins (EPPs) is more strongly influenced by the environment [25].

### 2.2. Effects of Interactions between Gluten and Starch on CSB Quality

Starch can interact with gluten proteins through secondary bonds, thus forming a unique network structure during thermal processing [26]. The ratio between gluten and starch also has a significant effect on water absorption ability, pasting properties, rheological behaviors and the stability of the gluten–starch matrix, as well as the overall quality of CSB. Previous studies have been focused on the effects of protein and starch on CSB separately [9,27,28]. Recently, it was reported that an increase in gluten–starch ratio makes CSB exhibit a declining trend in hardness and chewiness [8]. Thus, it is essential to consider the balance between gluten proteins and starch in the research on CSB quality. The starch composition is another factor affecting CSB quality. It has been found that the relatively shorter shelf-life of CSB is mainly due to its higher moisture content than baked products. Hence, attempts have been made to tackle this problem by blending flour with certain amounts of waxy wheat flour [29,30,31]. Flour blended with 15% waxy wheat flour exhibited a reduction of staling without affecting the CSB quality thanks to changes in starch stability along with the adjustment of amylopectin content [30]. Blending 20–40% flour of Jinuo16, a waxy wheat variety, into flour of other wheat varieties was found to yield better taste, appearance and storage properties of flour products, including CSB [29].

### 2.3. Protein Content

There is a good linear relationship between protein content and bread quality [32,33]. However, wheat varieties with high protein content or strong dough strength do not necessarily result in high CSB quality. There is a reported case of negative correlations between protein or gluten strength and CSB total quality score in [34]. It was also found that excessive elasticity caused shrinkage and wrinkles in the final CSB products [35]. On the other hand, a significant positive correlation was observed between protein content and the total quality score of CSB when the flour protein was less than 10%. It is well established that flour with medium or medium-to-high protein content is more appropriate for CSB production [1,3,8,22,36]. Protein content and wet gluten content greatly contribute to the CSB texture properties of elasticity, cohesiveness and resilience [37].

The protein content of wheat varieties in China varies from 10.9% to 13.4% [38]. Different protein contents should be recommended for the production of different styles of CSB. A study of 71 wheat varieties showed that the desirable protein content and SDS sedimentation value for CSB production should be 10.2–14% and 12.5–23.2 mL, respectively [27]. For northern-style CSB, a study of 114 wheat varieties and 19 quality parameters recommended that the protein-related parameters for higher CSB quality should be a protein content of 10.7–12.7%, a wet gluten content of 29.4–36.0% and an SDS sedimentation value of 16.9–23.9 mL [39]. For southern-style CSB, Huang et al. [40] reported that protein content was correlated with the specific volume (r = 0.67, *p* < 0.001), and had a stronger effect on southern-style CSB than on northern-style CSB. Crosbie et al. [41] suggested that flours with protein content of 9.5–11.0% and medium gluten strength were more desirable for southern-style CSB production. For Guangdong-style CSB, a flour protein content of 7.5–8.0% and weak dough strength were recommended according to a study with seven Australian soft wheat varieties [5].

### 2.4. Glutenins

Glutenins consist of high- and low-molecular-weight glutenin subunits (HMW-GS, LMW-GS), and confer dough viscoelasticity [42,43,44]. Glutenins account for only about 10% of the total storage protein in wheat grains; however, they have been long considered a major determinant of dough quality [45,46].

HMW-GSs are encoded by the *Glu-1* loci on wheat chromosomes, with each locus encoding two types of HMW-GS, namely the x- and y-type [47,48,49]. Abundant allelic variations of HMW-GS have been identified so far [50,51,52]. For the quality of bread, subunits 1 or 2* at *Glu-A1*, 7+8, 17+18, 13+16 and 14+15 at *Glu-B1*, and 5+10 at *Glu-D1* are considered as superior subunits [42,53]; however, this is not entirely true for the quality of CSB. Contradictory results have been reported, and no solid conclusions can be made from the literature (Table 1). Several studies have shown that the CSB quality score has positive correlations with subunits 5+10 at the *Glu-D1* locus [9,54,55]. Jin et al. [56] used 25 near-isogenic lines to study the effects of different allelic variations of HMW-GS and LMW-GS on the quality of northern-style CSB, and found that the lines with 1, 7+9 and 2+12 could achieve the highest score of northern-style CSB. Ma and Byung-Kee [30] reported that HMW glutenin subunits 1 or 2*, 7*+8 and 5+10 could contribute to higher CSB scores than their counterpart allelic variations. Zhang et al. [57] suggested that the lines with the HMW glutenin subunit composition of 2*, 17+_, 5+_ and 2*, 17+_, 2+12 could produce CSB with better quality. These differences can be mainly attributed to the fact that good CSB quality requires moderate protein content and dough strength. Therefore, both protein content and dough strength are required to be simultaneously considered in the breeding of wheat varieties for CSB production. Varieties with high protein content should be incorporated with HMW-GSs that confer weak dough strength and vice versa.

Compared with those of HMW, the effects of LMW glutenin subunits on CSB quality have received less attention, probably due to their less-important roles in dough strength, even though the ratio of HMW-GS to LMW-GS in wheat is about 1:3. An increase in the ratio of HMW-GS to LMW-GS will result in larger glutenin polymers, which can contribute to better loaf quality [58]. LMW-GSs are essential contributors of dough extensibility [59]. Similar to HMW-GSs, abundant allelic variations of LMW-GSs also exist in wheat and durum germplasm [60,61,62,63]. Jin et al. [56] reported that *Glu-A3e*, *Glu-B3b* and *Glu-D3c* could contribute to the highest total score of northern-style CSB.

Wheat gluten contributes to 40–60% of baking quality [44]. Most genetic studies of wheat end-use quality dissect the effects of glutenin alleles including HMW- and LMW-GSs, which have been efficiently guiding wheat quality improvement through breeding. However, for CSB quality, apart from the 5+10 subunits that are considered to be favorable allele/subunits, the other alleles’ genetic effects are contradictory (Table 1). Genetic studies of LMW-GS alleles are seldomly carried out for CSB quality, demonstrating a direction of future genetic study. In addition, few studies have been carried out concerning whole-genome surveys including QTL mapping or GWAS studies for genetic factors underlying CSB quality [64,65,66], and more studies using expanded genotypes are essential.

### 2.5. Gliadins

Gliadins are a mixture of heterogeneous monomeric proteins with molecular weight ranging from 3 to 8 × 10^4^ Da. They contribute to the foaming and viscous properties of dough [67]. According to their biochemical and genetic characteristics, gliadins can be further categorized into ω-, α-, β- and γ-type subgroups. Among them, ω-gliadins have higher molecular weight (ranging from 44,000 to 80,000 Da) than α- and γ-gliadins (ranging from 31,000 to 35,000 Da) [68]. Some studies have shown that γ-gliad ins are positively associated with quality parameters such as the Zeleny sedimentation value and loaf volume, while ω-gliadins are widely considered to be responsible for poor bread quality [69,70]. The α- and γ-gliadins are sulfur-rich, while the ω-gliadins are sulfur-poor. The presence of hydrophilic and hydrophobic parts in α- and γ-gliadins confers them with an amphiphilic property [67]. Different gliadin proteins tend to be inherited together as blocks [71], and the expression of these gliadins was found to be controlled by genes on homologous groups 1 and 6 in wheat, which are designated as *Gli-A1, Gli-B1, Gli-D1, Gli-A2, Gli-B2* and *Gli-D2* [72,73].

Lines with 1B/1R translocation were found to contain high levels of ω-gliadins and low levels of LMW-GS. The 1B/1R translocation was reported to have a negative effect on the bread quality [38,47,74]. For CSB production, non-translocation lines also showed higher specific volume, better crumb structure and total score [75]. However, a study involving 33 varieties from China, Mexico and Australia on the association between gluten proteins and the quality of northern-style CSB showed that there was no significant difference between the translocation and non-translocation lines [48].

**Table 1:** Technical parameters and requirements for better CSB quality.

CSB Style	Flour Protein Content %	Dough Strength	HMW-GS	LMW-GS	Gliadin	Additives
Northern	10.7–12.7 [39]	Medium to high [39]	5+10 [54]; 1, 7+9, 2+12 [56]; 1 or 2*, 7*+8, 5+10 [75]; 2*, 17+_, 5+10 or 2+12 [10].	Higher HMW-GS to LMW-GS ratio favorable [58]; favorable alleles: Glu-A3e, Glu-B3b, Glu-D3c [56].	Lower gliadin-to-glutenin ratio favorable [48].	Xylanase [76]; sodium alginate, emulsifiers, lipids etc. [3]; waxy flour [30].
Southern	9.5–11.0 [41]	Medium [41]	5+10 [54]; 1 or 2*, 7*+8, 5+10 [75]; 2*, 17+_, 5+10 or 2+12 [10].	Higher HMW-GS to LMW-GS ratio favorable [58].	Lower gliadin-to-glutenin ratio favorable [48].	Sodium alginate, emulsifiers, lipids, etc. [3]; waxy flour [30].
Guangdong	7.5–8.0 [5]	Weak [5]	5+10 [54]; 1 or 2*, 7*+8, 5+10 [75]; 2*, 17+_, 5+10 or 2+12 [10].	Higher HMW-GS to LMW-GS ratio favorable [58].	Lower gliadin-to-glutenin ratio favorable [48].	Sodium alginate, emulsifiers, lipids etc. [3]; waxy flour [30].

Even though glutenins are known to play a key role in gluten strength, gliadins are also indispensable as they interact with glutenins and influence the formation of GMP [60,61]. A higher proportion of gliadins usually leads to a weaker dough strength [77], which is in fact more desirable in CSB production. Nevertheless, the balance between gliadins and glutenins should not be ignored, as Zhang et al. [48] reported that the gliadin–glutenin ratio has significant and negative correlations with CSB quality.

## 3. Rheological Parameters Related to CSB Production

### 3.1. Genetic Studies and Prediction Model on CSB Quality

The production of wheat flour into various end products largely depends on the rheological properties of the wheat dough. The rheological properties of gluten are deemed more important than the total protein content [33]. To evaluate those properties, several instruments have been designed to assess either the mixing or stretching characteristics of dough. The development of such instruments, including the Extensograph [78], Mixograph [79], Farinograph [80] and Alveograph [81], allows more accurate and objective evaluation with large throughput and smaller flour samples compared with empirical procedures. Based on the phenotype data that have been obtained, genetic studies on CSB quality such as QTL mapping [64,65,66,82] have been conducted. The major QTL regions determining CSB quality are Xgwm111-Xgdm14-6D and Xgwm210-Xwmc382.2-2B on chromosome 2B [64,65], Xgwm644-Xgwm193-Xgwm608b on 6B, Xissr23b-Xwmc308-Xsrap7c on 4A [82], RFL_CONTIG2160_524-WSNP_CAP12_C2438_1180601 on 1A and EX_C101685_705-RAC875_C27536_611 on 4B [82]. An analysis of 75 spring bread wheat cultivars/lines with different puroindoline alleles found that the cultivars/lines with *Pinb-D1b* exhibited significantly higher total CSB quality scores than those with *Pina-D1b* or the wild type [83].

During a typical CSB production process, the flour, yeast and water are first mixed into a dough, followed by dough expansion due to the gas released by the yeast during the fermentation. Then, thin skin and soft crumb are formed after the steaming of the dough [84]. Considerable changes in rheological properties occur during these processes. To evaluate the performance of different varieties and the effects of various factors on CSB production, a number of studies have used different instruments for comparing the rheological behaviors of the dough during CSB production in the last three decades. Based on an analysis between flour quality parameters and CSB quality, Huang et al. [40] established a model for predicting the total CSB score: total score = 56.19–29.499 × ash content (%) + 0.047 × maximum resistance (BU) + 0.055 × peak viscosity (RVU) (R^2^ = 0.54***). Where grade A is score of 75 and over, grade B is between 65 and 75 and grade C is below 65. Ma and Byung-Kee [75] proposed a different model using other parameters: total score = 42.647 + 0.328 × SDS sedimentation volume (mL) + 0.477 × wet gluten content (%) + 3.778 × midline peak time (min). These models are very useful for the establishment of a universally recognized benchmark for the standardized production of CSB. So far, there have been very few studies in this area. Further research is needed to use more instruments and parameters with the incorporation of more wheat samples from different genetic backgrounds to enhance the accuracy of these prediction models.

### 3.2. Extensograph

The Extensograph is an ideal instrument for the measurement of the stretching properties of dough. In an Extensograph test, the dough is subjected to both shear and uniaxial extension force, and two major parameters, maximum resistance (EU) and extensibility (mm), are measured [85]. The Extensograph has been proven very useful in the quality prediction of wheat flour [86,87]. It has been reported that the total CSB score has no significant correlation with maximum resistance, while it has a positive correlation with extensibility [1]. Yue et al. [84] reported that wheat varieties with higher maximum resistance (*p* ≤ 0.05) have higher specific volume of CSB. Zhang et al. [24] demonstrated that the proportion of UPP is positively correlated with the maximum resistance with r = 0.90, while EPP is positively correlated with the extensibility. Additionally, the extension ratio was also found to influence the elasticity of northern-style CSB [36].

### 3.3. Mixograph

The Mixograph was initially developed to study the action of high-speed commercial mixers [88]. A minimum flour sample of two grams is required, and over 40 parameters can be generated by the Mixograph [79]. It records the resistance of dough to mixing during the mixing process, and thus estimates and predicts the dough strength [89,90]. Mixograph parameters including Mixograph absorption, midline peak time and midline peak value were all found to be positively correlated with the total CSB score, regardless of the protein content [30,91]. Therefore, they are considered effective predictors of CSB production quality. A study of 19 wheat varieties with protein contents of 6.6–9.9% to evaluate the suitability of soft red winter wheats for CSB production indicated that midline peak time is significantly associated with the total CSB score and 89% of the variation in total score could be attributed to SDS sedimentation, wet gluten content and midline peak time [75].

### 3.4. Farinograph

The Farinograph evaluates the resistance of dough by recording its behavior upon mixing at a constant speed with specified water addition. Parameters including water absorption, development time, stability time, mixing tolerance index, Farinograph quality number and Farinograph unit are determined in a Farinograph test [80]. The stability time was found to be positively correlated with the proportion of UPP (r = 0.78) [24] and resilience [36]. He et al. [1] evaluated 66 samples with protein content range of 9.6–16.2% and demonstrated that the total CSB score had a positive correlation with the stability time, while it had a negative correlation with the development time. However, Zhu et al. [92] reported a negative correlation between the stability time and CSB quality in Australian wheats, and a positive correlation in Chinese wheats based on 17 samples with protein content range of 7.5–13%. The recommended Farinograph and Extensograph parameters for CSB production are listed in Table 2.

### 3.5. Alveograph and Texture Analyzer

The Alveograph is used to determine the stretching of the dough as well as the tensile strength during dough stretching. Three major parameters are generated from an Alveograph test, including P (height of the curve), which measures the tenacity of the dough; L (length of the curve), which measures the dough extensibility; and W, which is a computed value that reflects the resistance to extension and total gluten strength [81]. A study of 31 commercial flour products indicated that the desirable Alveograph values for good northern-style CSB should be P = 72–110, L = 55–70 and W = 140–210. Alveograph parameters showed that strong w/fleo90ILOLgluten wheats are not suitable for northern-style CSB, while wheat flour with good extensibility is more desirable [94].

Previously, the eating and texture properties of CSB have been evaluated empirically [39]. By using texture profile analysis (TPA), texture parameters such as hardness, resilience, cohesiveness, elasticity and chewiness of CSB can be evaluated in a standardized manner [36]. A TPA test of 20 commercial samples of southern-style CSB showed that texture quality parameters could be used to predict the total CSB score, and the hardness and cohesiveness determined by TPA could account for a significant proportion of variance in CSB quality [95].

## 4. Effects of Food Additives and Frozen Storage on Gluten Proteins and CSB Quality

### 4.1. Improvements of CSB Quality with Dietary Fiber and Food Additives

In recent years, the increase of health awareness has promoted the trend of adding wholemeal flour of wheat and other cereals in CSB production to improve its taste and nutritional value. Studies on foxtail millet flour, quinoa flour, buckwheat flour, finger millet and red kidney bean flour, rice flour and rye flour have been documented [23,96]. However, additions of wheat bran and other dietary fibers often cause gluten dehydration and the destruction of gluten structure integrity, leading to deteriorations in the gluten network, appearance and sensory characteristics of CSB [97]. Hence, during commercial production, several hydrocolloids are usually used to improve the overall quality parameters, including softness, specific volume, brightness, and to prolong the shelf-life of CSB [46,98]. The possible reason is that these ingredients could hinder gluten aggregation and enhance the CO_2_- and water-holding capacity of dough [46]. Enzymes are also commonly used as a processing aid to modify the rheology and processing properties of dough. Northern-style CSB modified by xylanase showed higher specific volume and lower hardness in [76]. Wheat flour with the addition of xylanase, GOX and cellulase had increased GMP and significantly increased water-extractable arabinoxylan content (*p* < 0.05), and consequently a more compact and continuous gluten matrix [99]. Additionally, a number of other additives have been used to extend the shelf-life of CSB, including sodium alginate, emulsifiers, lipids etc. [3].

### 4.2. Effects of Frozen Storage and Improvements

Advantages in centralized manufacturing and distribution processes have driven growing market interests in frozen bakery goods, due to the long shelf-life required during storage and transportation [100]. However, frozen storage leads to a decline in the dough quality of CSB, such as a decrease in specific volume and increase in hardness [101], which is mainly attributed to the loss of gas retention [102], yeast activity [103] and gluten network integrity [104], as well as the formation or recrystallization of ice crystals [105]. There is also an obvious increase in the depolymerization of GMP during frozen storage, along with an increase in the amount of SH groups in the dough [106,107], which is also a major factor leading to the quality deterioration of frozen dough and the increased hardness of CSB. This depolymerization of GMP is caused by the reduced incorporation of glutenin in glutenin–gliadin crosslinking [60] and a considerable loss in the number of HMW glutenins [61]. At the peptide level, gluten deterioration during frozen storage is due to the depolymerization of GMP resulting from the interruption of inter-chain SS bonds and the conversion of α-helix and β-turn to β-sheet structures [108]. As a more ordered structure, α-helix is regarded as the backbone of polypeptides [109]. Therefore, a reduction of this structure reflects a more disordered gluten network.

Several attempts have been made to mitigate the negative effects of frozen storage on CSB dough quality. Doughs made from flours with protein contents of 9.5–11% were found to have better resistance to the damages caused by the freeze–thaw process and maintained a CSB quality score close to that of fresh dough [101]. Oat β-glucan could inhibit water migration and starch retrogradation, thus alleviating the quality deterioration and staling of CSB [110]. Replacement of yeast with chemical leaveners also resulted in an improved stability of CSB under frozen storage [108]. Frozen storage temperature was also found to influence the GMP depolymerization and CSB quality, with lower storage temperature resulting in a higher GMP content and CSB quality [111].

## 5. Other Factors

In addition to the above-mentioned main factors affecting CSB quality, there are numerous other factors affect the CSB product quality, including the composition and properties of basic ingredients such as wheat flour and yeast/starter [2,112]. Sourdough fermentation, as an ancient biotechnology, has traditionally been used in CSB production. Improvements in CSB crumb structure, flavor, nutritional value, shelf-life and specific volume have been reported using sourdough fermentation [113,114]. Both endogenous and exogenous lipids affect CSB quality. Defatted flours produced CSBs with similar specific volume but deteriorated crumb characteristics [115]. Adding peanut oil significantly increased CSB specific volume, texture, cell density and cell-to-total-area ratio, and outperformed commercial shortening and other plant-sourced oils [116]. Degree of debranning is a factor affects the sensory and textural aspects of CSB quality [6]. A higher degree of debranning had positive effects on the volume, volume-to-weight ratio, height and structure of CSB in [6]. Packaging temperature for room-temperature storage has impacts on the CSB quality. With the increase of packaging temperature from 50 °C to 90 °C, the hardness of CSB was decreased, while no significant difference in moisture migration during room-temperature storage was observed [117].

## 6. Conclusions

Chinese steamed bread has served as an indispensable staple food in China for over 1700 years, and it is gaining popularity globally. Because of the low incorporation of salt and oil and the addition of other dietary fibers, it can be categorized as a healthy food. Gluten proteins, especially glutenins and gliadins, are the major determinants of the overall quality of CSB. The protein content, gluten/starch ratio, glutenin/gliadin ratio and HMW-GS composition all play important roles in determining the rheological properties of wheat dough, and therefore the appearance and sensory quality of the CSB. Several instruments, including Extensograph, Mixograph, Farinograph, Alveograph and texture analyzer, have been used to evaluate the rheological characteristics of wheat varieties and their CSB quality, and recommended parameters have been proposed for CSB production. Parameters acquired from these instruments could facilitate the future perfection of prediction models for CSB quality, which will accelerate the genetic improvement and breeding process. Frozen storage has been increasingly used in the manufacturing and distribution process of CSB despite its negative effects on dough quality, mainly caused by GMP depolymerization. To alleviate the quality deterioration during frozen storage, several measures have been adopted, including the use of flour with higher protein content, food additives, lower storage temperature etc.

## Figures and Tables

**Table 2:** Recommended Farinograph and Extensograph parameters for CSB production.

Sample Size	CSB Type	Farinograph Parameters	Extensograph Parameters	Reference
		stability time ≥ 3.0 min for superfine grade; stability time ≤ 3.0 min for common grade		
71	Northern	development time: 2.2–7.3 min; stability time: 3.0–11.5 min		[27]
114	Northern	development time: 2.1–3.9 min; stability time: 1.4–4 min	maximum resistance 174–576 EU; extensibility 149–183 mm	[39]
25	Southern	stability time ≤ 2.0 min; water absorption ≤ 56%	maximum resistance 480~630 EU; extensibility ≤110 mm	[10]
10	Northern	development time: 2.2–4.0 min; stability time: 3.5–7 min	maximum resistance 260–420 EU; extensibility 130–210 mm	[93]

## Data Availability

No new data were created.
